# Brain natriuretic peptide in acute heart failure and its association with glomerular filtration rate: A systematic review and meta-analysis

**DOI:** 10.1097/MD.0000000000036933

**Published:** 2024-02-23

**Authors:** Hamdah Bashir Mughal, Ayesha Isani Majeed, Maria Aftab, Muhammad Furqan Ubaid, Sabahat Zahra, Muhammad Sajid Rafiq Abbasi, Mamoon Qadir, Mumtaz Ahmad, Amna Akbar, Sabahat Tasneem, Sarosh Khan Jadoon, Maham Tariq, Saddam Hussain, Shahad Saif Khandker, Sarosh Alvi

**Affiliations:** a Registered Medical Practitioner, Azad Jammu & Kashmir Medical College, Muzaffarabad, Azad Jammu and Kashmir, Pakistan; b Pakistan Institute of Medical Sciences, Islamabad, Pakistan; c Queen Elizabeth Hospital Birmingham, Birmingham, UK; d Tufts Medical Center, Boston, MA; e Acute and General Medicine, Queen Elizabeth Hospital Birmingham, Birmingham, UK; f Department of Nephrology, Pakistan Institute of Medical Sciences, Islamabad, Pakistan; g Head of Cardiology Department Fed Govt Polyclinic and Kulsum International Hospital, Islamabad, Pakistan; h Abbas Institute of Medical Sciences, Muzaffarabad AJK, Pakistan; i District Headquarter Hospital Jhelum Valley, Muzaffarabad AJK, Pakistan; j Health Services Academy, Islamabad, Pakistan; k CMH/SKBZ, AJK Muzaffarabad, Pakistan; l Gujranwala, Teaching Hospital, Gujranwala, Pakistan; m Punjab Institute of Neurosciences, Lahore, Pakistan; n Gonoshasthaya-RMA Biotech Limited, Savar, Bangladesh; o Teaching Faculty, University of Khartoum, Khartoum, Sudan.

**Keywords:** acute heart failure (AHF), brain natriuretic peptide (BNP), glomerular filtration rate (GFR), prognostic biomarker

## Abstract

**Background:**

Acute heart failure (AHF) is one of the most common cardiovascular diseases. Early diagnosis and prognosis are essential, as they can eventually lead to a fatal condition. Recently, brain natriuretic peptide (BNP) has been recognized as one of the most popular biomarkers for AHF. Changes in glomerular filtration rate (GFR) are often observed in AHF.

**Methods:**

We searched PubMed, Google Scholar, and ScienceDirect between March and June 2023. Original case control studies written in English that assessed levels oh BNP in AHF were included. Systematic reviews, letters to editor, correspondence, comprehensive reviews, and duplicated studies were excluded. Funnel plots were constructed to assess publication bias.

**Results:**

A total of 9 studies were selected and we obtained the mean difference (MD) of BNP level to be 2.57 (95% CI: 1.35, 3.78), and GFR to be −15.52, (95% CI: −23.35, −7.70) in AHF patients. Sensitivity analyses supported the robustness of the outcome.

**Conclusion:**

Results indicated that BNP was a promising prognostic biomarker of AHF, whereas GFR was found to be negatively correlated with AHF.

## 1. Introduction

Acute heart failure (AHF) is the sudden onset or aggravation of signs and symptoms of heart failure caused by a rapid increase in intra cardiac filling pressure or myocardial failure.^[1]^ A prevalence of 1% to 2% of AHF has been reported in the adult population in developed countries, it affects approximately 64 million people around the globe.^[2,3]^ Impaired function of the ventricles, pericardium, blood vessels, or heart valves alone or in combination may be responsible for AHF. In addition, over-stimulation of neurohumoral function, ventricular remodeling, ischemia-related dysfunction, excessive hemodynamic load, abnormal calcium cycle in myocytes, hyperactive apoptosis, over-proliferation of the extracellular matrix, and gene mutations can be critically related to AHF.^[3–5]^ AHF is an emergency condition that can be fatal; therefore, an early diagnosis is crucial. Brain Natriuretic Peptide (BNP, also known as B-type natriuretic peptide) and cleaved inactive N- terminal fragment of the BNP precursor (N-terminal proBNP) are important biomarkers that can be used to evaluate patients with heart failure.^[5]^ Secretion of BNP, which has an antagonistic effect against angiotensin II, increases during acute heart failure due to stress in the ventricles^[6]^ and resists volume overload.^[7]^

BNP has natriuretic, diuretic, hemo-concentrating, and vasodilating properties, which are linked to inhibition of the renin-angiotensin-aldosterone system (RAAS) and sympathetic nervous system (SNS). Natriuretic peptides (NPs) help to maintain homeostasis during the early stages of AHF. However, as cardiac function gradually deteriorates, NPs lose effectiveness due to the inhibition of downstream signaling pathways, reduced production, and increased excretion or enzymatic degradation. An increase in NP occurs in response to an increase in the RAAS and SNS effects. Nevertheless, elevated plasma levels of NP are commonly observed in AHF. Thus, they are quantifiable indicators of the severity and prognosis of AHF and may help evaluate treatment efficacy in cardio renal syndrome.^[8]^

The heart and kidneys are vital organs that significantly affect each other. Renal perfusion is maintained by the differences in arterial and venous outflow pressures. Acute heart failure can lead to reduced cardiac output, which, in turn, lowers renal blood flow. This low renal perfusion causes the release of renin and thus activates the renin-angiotensin-aldosterone pathway. As a result, there is salt and water retention, as well as an increase in vascular tone, both of which serve to keep renal perfusion steady. However, excessive RAAS activation leads to deterioration of renal function by reducing pre-glomerular blood flow and increasing central venous pressure.^[9]^ The enhancement of systemic vascular resistance, increase in afterload and reduction in cardiac output further reduce renal blood flow, followed by a decreased glomerular filtration rate (GFR).^[10]^

To the best of our knowledge, no meta-analysis has assessed the mean difference (MD) of BNP and GFR in AHF based on case-control studies. Therefore, in this meta-analysis, we assessed the MD value of BNP with a 95% CI by comparing case-control data to assess whether it can be a crucial biomarker in the diagnosis and prognosis of AHF patients. In addition, we analyzed the MD value of the GFR to determine its association and significance in patients with AHF and to determine if this can be further assessed as another marker of AHF.

## 2. Methodology

### 2.1. Search strategy

Three databases (i.e., Google Scholar, PubMed, and ScienceDirect) were searched with specific keywords such as “natriuretic,” “brain natriuretic peptide,” “BNP,” “N-terminal proBNP,” “heart failure” and “acute heart failure.” Articles were searched in PubMed as well as ScienceDirect with an “advanced” mode, followed by “Title and abstract” and “Title, abstract, keywords,” respectively. In Google Scholar, articles were searched using “allintitles.” Boolean operators were used for different database searches. The search strategy is presented in Table S1, Supplemental Digital Content, http://links.lww.com/MD/L477

### 2.2. Eligibility criteria

Rigorous and specific searches were performed for articles on acute heart failure and BNP, using specific keywords and search strategies. However, because of the large number of articles, we excluded systematic or comprehensive reviews, correspondences, letters, and other articles that did not align with our interests. Duplicate articles were removed from the different databases. Only articles written in English were included in this study. After excluding these articles, we selected original case-control studies that assessed BNP levels in patients with AHF.

### 2.3. Quality assessment

Nine questions were answered in order to assess the quality of the included studies. The questions were obtained from the Study Quality Assessment Tools, the National Institutes of Health, and Systematic Reviews. Step 6: Assess the Quality of Included Studies (UNC).^[11,12]^ The score was given as the individual score for different answers (yes = 1, no = 0, partially = 0.5, not reported = no score) depending on each question. The total scores of 5, 6, and 8 were classified as low-scoring studies (high risk of bias), moderate-scored studies (moderate risk of bias), and high-scoring studies (low risk of bias), respectively. Funnel plots were constructed to assess publication bias (Fig. 2).

**Figure 1.**
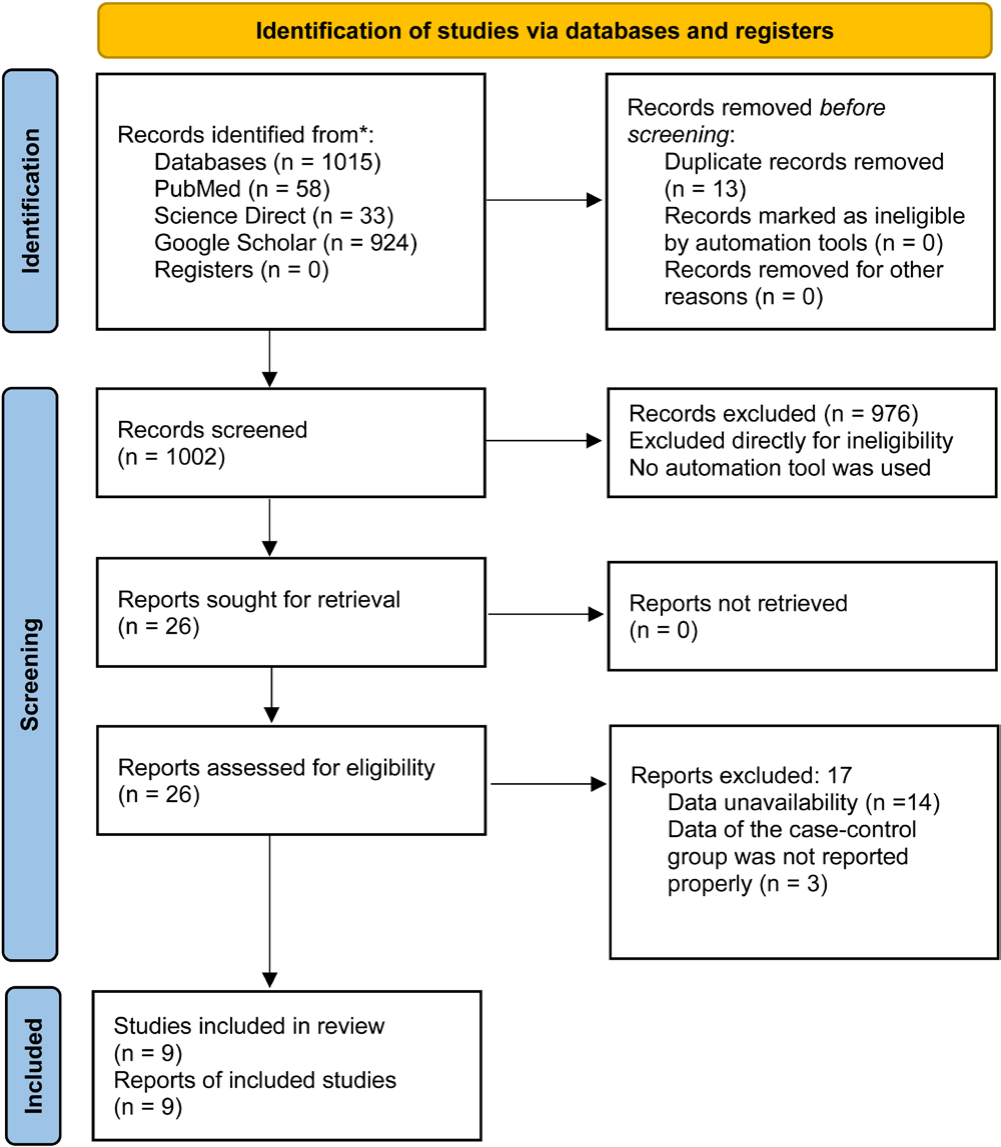
PRISMA flow diagram. PRISMA = the preferred reporting items for systematic reviews and meta-analyses.

### 2.4. Data extraction

Based on the eligibility criteria, data extraction was performed carefully from the included studies and independently by the authors. Several pieces of data and information were extracted as major characteristics of the included studies. Data for the meta-analyses i.e., mean ± standard deviation (SD) of all participants (case or control) were extracted separately from all studies that fulfilled the eligibility and inclusion criteria for the meta-analysis. EndNote software (version X8) was used for data extraction.

### 2.5. Data analyses

A random-effects model of mean difference (MD) with 95% confidence intervals (CIs) was used to analyze the differences in BNP levels between cases and controls among the included studies. Second, GFR data from the same studies were investigated and extracted for meta-analysis, in addition to BNP. The heterogeneity of the included studies was determined using *I*^2^ statistics (*I*^2^ > 75% implies substantial heterogeneity). RevMan (version-5.4) software was used for the meta-analysis.

### 2.6. Sensitivity analyses

To investigate the robustness of the results, sensitivity analyses were performed using different modes of analysis following a previous study with slight modifications. Primarily, a random-effects model was used to identify the sensitivity, excluding the slightly outlier studies used for BNP. Furthermore, a funnel plot was constructed using both random- and fixed-effects models using the same non-outlier studies. A forest plot was constructed using a fixed-effects model, with studies used to identify the mean difference in the GFR.

## 3. Results

### 3.1. Inclusion of studies

After the initial search, 1015 articles were identified from 3 online databases (PubMed, ScienceDirect, and Google Scholar). From all the initially found articles (n = 1015), 976 were immediately excluded because they were case reports, review articles, correspondence, letters, or original articles working with animal models, in vitro*, or in silico* experiments, other than full-length research articles working with human case-control models. Of the remaining 39 articles, 13 were excluded due to duplication. After excluding 17 articles that did not match our focus and study criteria (i.e., including different types of controls, lack of data, assessment of other diseases, etc.), 9 articles were included in this meta-analysis (Fig. 1, Figure S1, Supplemental Digital Content, http://links.lww.com/MD/L475).

### 3.2. Assessment of quality and publication bias

**Figure 2.**
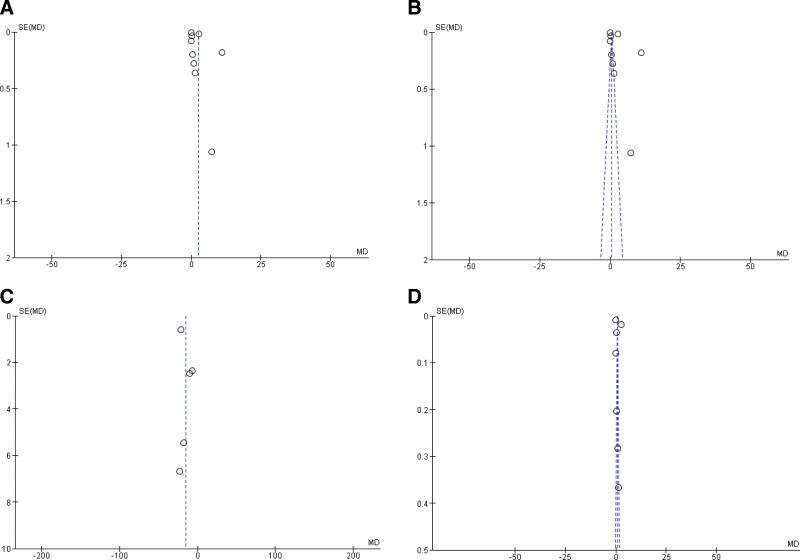
Funnel plot of the (A) overall included studies (n = 9) using random effect model and (B) fixed effect model used for overall BNP mean difference measurement. Again, funnel plot of the (C) studies (n = 5) with GFR data using random effect model and (D) fixed effect model used to determine the mean difference of GFR. BNP = brain natriuretic peptide, GFR = glomerular filtration rate.

Quality assessment of the studies determined that all included studies were of moderate (moderate risk of bias) or high quality (low risk of bias) (Table 1). Funayama (2011) and Johansson (2022) obtained the lowest score (6.5, moderate risk of bias), followed by Girerd (2022), Sultana (2010), Sutter (2015), and Yu (2017), who achieved scores of 7 among the included studies.^[13–18]^ Chetran 2022, Kang 2017, and Kanukurti 2020 achieved the highest score (8, low risk of bias).^[19–21]^ None of the studies had a score ≤ 5, indicating a high risk of bias. A funnel plot (Fig. 2) confirmed the absence of significant publication bias.

**Table 1:** Quality assessment of the included study.

Study ID	1	2	3	4	5	6	7	8	9	Overall score
Funayama 2011	Y	Y	Y	Y	Y	P	N	Y	NR	6.5
Girerd 2022	Y	Y	Y	Y	Y	Y	N	Y	NR	7
Chetran 2022	Y	Y	Y	Y	Y	NR	Y	Y	Y	8
Johansson 2022	Y	Y	Y	Y	Y	P	N	Y	NR	6.5
Kang 2017	Y	Y	Y	Y	Y	NR	Y	Y	Y	8
Kanukurti 2020	Y	Y	Y	Y	Y	NR	Y	Y	Y	8
Sultana 2010	Y	Y	Y	NR	Y	NR	Y	Y	Y	7
Sutter 2015	Y	Y	Y	N	Y	NR	Y	Y	Y	7
Yu 2017	Y	Y	Y	N	Y	NR	Y	Y	Y	7

1. Was the research question appropriate? 2. Was the study population clearly defined? 3.Were controls selected from the same or similar population including the same timeframe? 4. Did the author followed proper inclusion and exclusion criteria regarding case-control selection? 5. Were the cases clearly defined and differentiated from controls? 6. Were the assessors of exposure/risk blinded to the case or control status of participants? 7. Were the methods of quantity determination clearly defined? 8. Did authors use statistical analyses? 9. Were the measurements/tools valid to conduct the study? Y = Yes (Score = 1), N = No (Score = 0), P = Partially (Score = 0.5), NR = Not reported (No score)

### 3.3. Study characteristics

Major characteristics, such as study location, study design, condition of case and control, demographics (i.e., age and male-female ratio in percentage) of the study participants, and method of BNP measurement were extracted from each of the included studies. In addition to the exact BNP measurement methods used in Funayama 2011, Girerd 2022, and Johansson 2022, all information regarding the study characteristics was identified (Table 2).

**Table 2:** Major characteristics of the include study.

Study ID	Study type	Location	Participants Characteristics					BNP measurement method	References
			Male (%)	Female (%)	Age	Case	Control		
Funayama 2011	Case-Control	Japan	62.2	37.8	68.5 ± 10.5	HF	Healthy	NR	^[8]^
Girerd 2022	Rct	USA	76	24	67.55 ± 9.95	HF	HF re-hospitalization with lower symptoms	NR	^[9]^
Chetran 2022	Case-Control	Romania	63.8	36.3	65.18 ± 12.98	AHF	ambulatory without HF	CL-EIA	^[14]^
Johansson 2022	Case-Control	Sweden	36.3	63.7	78.3 ± 5.1	HFpEF	Healthy	NR	^[10]^
Kang 2017	Case-Control	China	35.4	64.6	64.05 ± 10.35	SHF	No SHF	ECLIA	^[15]^
Kanukurti 2020	Cross-Sectional Study	India	52	47	57 ± 0.165	HFpEF	Healthy	ECLIA	^[16]^
Sultana 2010	Case-Control	Bangladesh	56	44	39 ± 30	HF	Healthy	MEIA	^[11]^
Sutter 2015	Cohort Case-Control	Belgium	100	0	50 ± 3	HF/CHD	Free from coronary event	ECLA	^[12]^
Yu 2017	Case-Control	China	61.7	38.3	63.65 ± 7.85	CHF	Healthy	ELISA	^[13]^

CHD = coronary heart disease, CHF = coronary heart failure, CL-EIA = chemiluminiscence enzyme immune assay, ECLA = electrochemiluminiscence assay, ECLIA = electrochemiluminescence immunoassay, ELISA = enzyme-linked immunosorbent assay, HFpEF = heart failure with preserved ejection factor, MEIA = microparticle enzyme immunoassay, NR = not reported, SHF = systolic heart failure.

### 3.4. Main outcome

**Figure 3.**
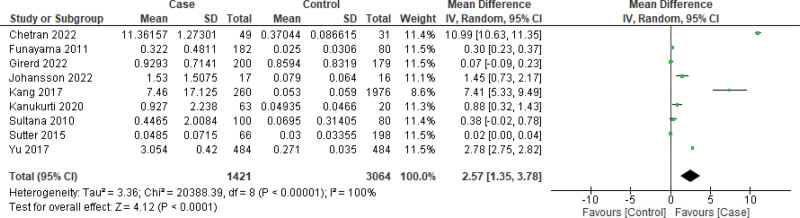
Forest plot of the included studies (n = 9) using random effect model to assess the overall mean difference of BNP. The unit of mean ± SD value was ng/mL. BNP = brain natriuretic peptide.

**Figure 4.**
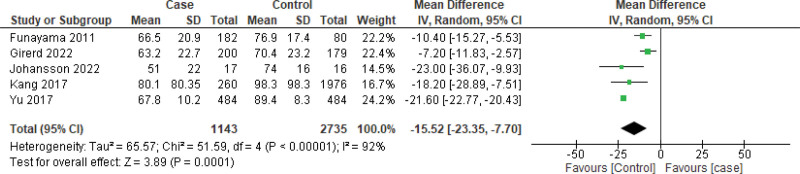
Forest plot of the studies (n = 5) to assess mean difference of GFR using random effect model. The unit of mean ± SD value was mL min^−1^ 1.73 m^−2^. GFR = glomerular filtration rate.

Primarily, the forest plot including all the studies (n = 9) (Fig. 3) demonstrated that the mean difference (MD) of BNP levels was 2.57, 95% CI: 1.35, 3.78, (*P* = .0001), favoring the case as compared to the control. Additionally, based on the availability of data, 5 studies were included in the mean difference in the GFR analysis. The MD for GFR was −15.52 (95% CI: −23.35, −7.70, *P* = .00001), which was found to favor the control as compared to the case group. The determined *I*^2^ > 98% for the analysis implied that the heterogeneity of the included studies was significantly high for BNP level assessments. However, for the MD measurement of the GFR, the heterogeneity (*I*^2^ = 92%) was determined to be lower as compared to that of BNP (Fig. 4).

### 3.5. Sensitivity analysis

**Figure 5.**
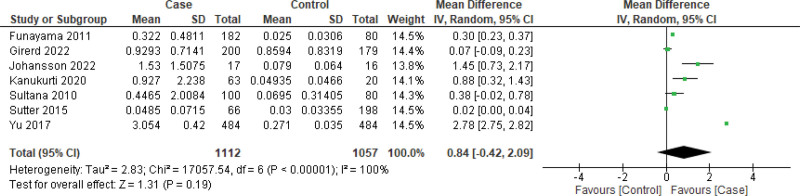
Forest plot of the studies excluding outlier studies (n = 2) to assess mean difference of BNP using random effect model. BNP = brain natriuretic peptide.

**Figure 6.**
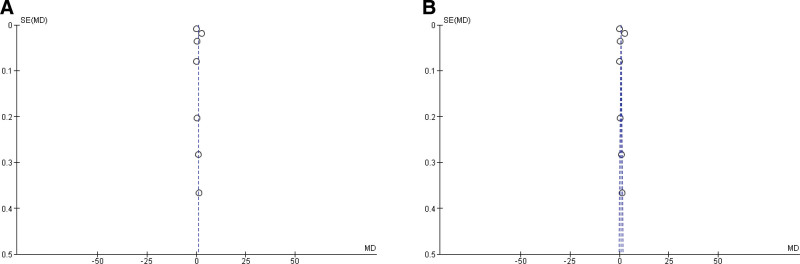
Funnel plot of the studies (For BNP assessment) excluding the outlier studies (n = 2) using (A) random effect model and (B) fixed effect model. BNP = brain natriuretic peptide.

**Figure 7.**
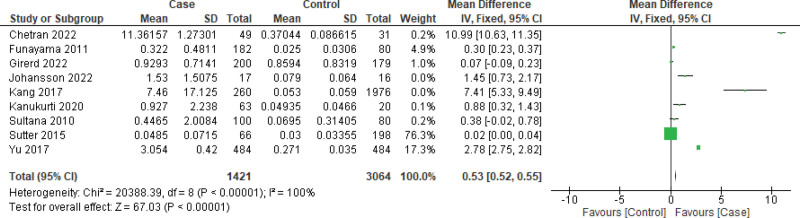
Forest plot of the overall included studies (n = 9) to assess mean difference of BNP using fixed effect model for sensitivity analysis. BNP = brain natriuretic peptide.

**Figure 8.**
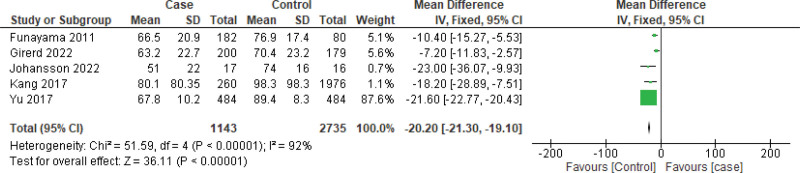
Forest plot of the studies (n = 5) to assess mean difference of GFR using fixed effect model for sensitivity analysis. GFR = glomerular filtration rate.

Owing to the high heterogeneity, we further analyzed the data for sensitivity, especially in the case of BNP measurements. First, we excluded 2 marginally outlier studies (i.e., Chetran 2022 and Kang 2017) to observe the impact (Fig. 5) and observed a similar result that favored this case (MD: 0.84 [95% CI: −0.42, 2.09]) as well. Funnel plots were constructed using both random- and fixed-effects models, which showed no significant outliers. (Fig. 6) Following the reconstruction of the forest and funnel plot excluding the outlier studies, a fixed effect model was used to assess the mean difference with all the included studies (n = 9), and as a result, MD: 0.53 [95% CI: 0.52, 0.55] was obtained, which also favored the case. In the case of GFR, the funnel plot did not display any considerable study bias; therefore, no further reconstruction of the forest or funnel plot was done, excluding any study. However, with the included studies (n = 5), the GFR forest plot was constructed using a fixed effects model (Figs. 7 and 8), and an MD of −20.20 [95% CI: −21.30, −19.10] still favored the control as the main outcome. These analyses further support the strength of our main outcomes.

## 4. Discussion

The primary precursor of BNP is 134-amino- acid-based pre-proBNP, which is cleaved and converted to pro-BNP. It is biologically processed and ultimately produces both an inactive N-terminal pro-BNP (NT-proBNP) fragment and an activated BNP fragment.^[22]^

AHF is a fatal condition requiring hospital admission.^[23]^ A preexisting comorbidity worsens the prognosis of heart failure.^[4]^ The presence of comorbidity can potentially lead to overestimation (renal failure, hyperthyroidism, inflammation, atrial fibrillation, old age, and female sex) or underestimation (obesity, pericardial effusion, and recent acute coronary syndrome) of BNP levels. Macro-proBNPemia and the use of sacubitril/valsartan can lead to overestimation of BNP.^[24]^ Guidelines by the “European Society of Cardiology” must be followed when measuring BNP levels.^[25]^ According to previous research, patients with AHF and acute dyspnea can be specifically identified by measuring BNP levels. The BNP level of 100 pg/mL or 0.1 ng/mL has a 90% negative predictive value, and 500 pg/mL or 0.5 ng/mL has an 81% positive predictive value.^[26]^ The highest negative predictive value reported for NP was 94% to 98%.^[27]^ Our findings strongly supported this correlation. Moreover, the BNP level is a strong predictor of the risk of death and cardiovascular events in patients previously diagnosed with heart failure or cardiac dysfunction.

As mentioned earlier, the activation of neurohumoral mechanisms (RAAS and SNS) results in decreased cardiac output, which in turn causes a decrease in renal blood flow and an increase in central venous pressure, both of which ultimately cause renal injury. Adverse drug reactions caused by diuretics, particularly in heart failure, also result in renal damage.^[28]^

An increase of ≥0.3 mg/dL in serum creatinine or decrease of ≥25% eGFR (mL/min/1.73m^2^) compared to baseline values at admission (Modification of Diet in Renal Disease) can be defined as worsening of renal function (WRF).^[29]^ The implications of WRF during the early phase of AHF can be better understood by evaluating the decongestion process (measured as the percentage drop in BNP concentration).^[30]^ A percentage decrease of ≥30% in BNP levels when compared to admission values was shown to be associated with favorable outcomes, both during the hospital stay and at 1 year follow-up.^[31,32]^ A combination of BNP ≥ 250 pg/mL, occurrence of in-hospital WRF, and history of hospital admission for heart failure predicted composite outcomes (short-term and long-term cardiovascular mortality).^[33]^

Approximately 4.5% of patients with chronic kidney disease are usually observed to display an eGFR of 60 mL/min/1.73 m^2^, while more than 50% of chronic and acute heart failure patients show similar deterioration in eGFR.^[34]^ Recently, the prognostic value of a reduced GFR in patients with AHF has been recognized. Previous retrospective investigations of randomized controlled trials found a strong association between increased mortality rates and lower GFR.^[35,36]^ Our analysis also correlated with these findings, indicating that GFR measurements can play an important role in the prognosis of AHF.

## 5. Conclusion

Our analyses suggest that careful measurement of BNP levels may be vital for AHF diagnosis and prognosis. In addition to the strong negative correlation between AHF severity and GFR, GFR can also be a vital indicator of AHF. However, simultaneously measuring both parameters can certainly be helpful in ensuring the condition of a patient with AHF.

## Acknowledgments

Direct technical help in the form of statistics/data manipulation was provided by Google Scholar, PubMed, and Science Direct: Furthermore, and indirect assistance was provided by SciFinder® and Sci-hub.

## Author contributions

**Conceptualization:** Ayesha Isani Majeed, Mamoon Qadir, Mumtaz Ahmad.

**Data curation:** Hamdah Bashir Mughal, Maria Aftab, Muhammad Furqan Ubaid, Sabahat Zahra, Amna Akbar, Sarosh Khan Jadoon, Saddam Hussain

**Formal analysis:** Shahad Saif Khandker

**Methodology:** Muhammad Sajid Rafiq Abbasi, Amna Akbar, Saddam Hussain

**Resources:** Ayesha Isani Majeed, Mumtaz Ahmad.

**Software:** Sarosh Alvi

**Supervision:** Ayesha Isani Majeed, Mamoon Qadir

**Writing – original draft:** Hamdah Bashir Mughal, Sabahat Tasneem, Shahad Saif Khandker.

**Writing – review & editing:** Ayesha Isani Majeed, Amna Akbar, Maham Tariq, Sarosh Alvi.

## Supplementary Material




