# Supplementary material for: Brain natriuretic peptide in acute heart failure and its association with glomerular filtration rate: A systematic review and meta-analysis
Source: Medicine (Baltimore). 2024 Feb 23;103(8):e36933. doi: 10.1097/MD.0000000000036933 (PMC11309607; doi:10.1097/MD.0000000000036933)
Supplement: Supplementary file 1 [file medi-103-e36933-s001.docx]

**Supplementary table: Search strategy for this study**

| **Databases** | **Search strategy** |
| --- | --- |
| **Google scholar** | **allintitle: Natriuretic AND heart AND case control**  **allintitle: Bnp AND heart AND case control**  **allintitle: Bnp AND heart failure** |
| **PubMed** | **((((Brain natriuretic peptide[Title/Abstract]) OR (bnp[Title/Abstract])) OR (N-terminal proBNP (NT-proBNP[Title/Abstract]))) AND ((heart failure[Title/Abstract]) OR (acute heart failure[Title/Abstract]))) AND (case control[Title/Abstract])** |
| **ScienceDirect** | **Title, abstract, keywords: Brain natriuretic peptide heart failure case control**  **Title, abstract, keywords: Bnp heart failure case control** |
