# Supplementary material for: Brain natriuretic peptide in acute heart failure and its association with glomerular filtration rate: A systematic review and meta-analysis
Source: Medicine (Baltimore). 2024 Feb 23;103(8):e36933. doi: 10.1097/MD.0000000000036933 (PMC11309607; doi:10.1097/MD.0000000000036933)

**Figure 1:** PRISMA flow diagram.

PRISMA = the preferred reporting items for systematic reviews and meta-analyses


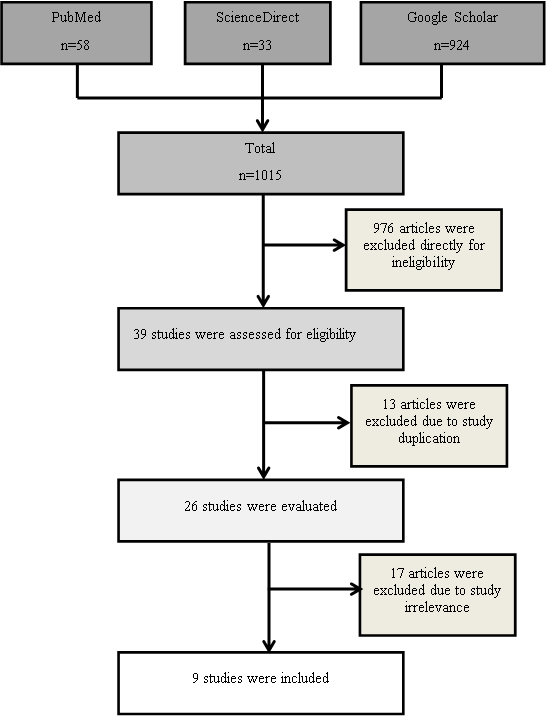

Supplement: Supplementary file 2 [file medi-103-e36933-s002.docx]
